# A Quantitative Comparison of Inhibitory Interneuron Size and Distribution between Mouse and Macaque V1, Using Calcium-Binding Proteins

**DOI:** 10.1093/texcom/tgaa068

**Published:** 2020-09-24

**Authors:** Roxana N Kooijmans, Wesley Sierhuis, Matthew W Self, Pieter R Roelfsema

**Affiliations:** Department of Vision & Cognition, Netherlands Institute for Neuroscience, 1105 BA, Amsterdam, the Netherlands; Institute of Neurosciences and Medicine (INM-1), Research Centre Jülich, 52425, Jülich, Germany; Department of Integrative Neurophysiology, CNCR, VU University, 1081 HV, Amsterdam, the Netherlands; Department of Vision & Cognition, Netherlands Institute for Neuroscience, 1105 BA, Amsterdam, the Netherlands; Department of Vision & Cognition, Netherlands Institute for Neuroscience, 1105 BA, Amsterdam, the Netherlands; Department of Vision & Cognition, Netherlands Institute for Neuroscience, 1105 BA, Amsterdam, the Netherlands; Department of Integrative Neurophysiology, CNCR, VU University, 1081 HV, Amsterdam, the Netherlands; Department of Psychiatry, Amsterdam UMC, 1105 AZ, Amsterdam, the Netherlands

**Keywords:** cross-species, interneurons, quantification

## Abstract

The mouse is a useful and popular model for studying of visual cortical function. To facilitate the translation of results from mice to primates, it is important to establish the extent of cortical organization equivalence between species and to identify possible differences. We focused on the different types of interneurons as defined by calcium-binding protein (CBP) expression in the layers of primary visual cortex (V1) in mouse and rhesus macaque. CBPs parvalbumin (PV), calbindin (CB), and calretinin (CR) provide a standard, largely nonoverlapping, labeling scheme in macaque, with preserved corresponding morphologies in mouse, despite a slightly higher overlap. Other protein markers, which are relevant in mouse, are not preserved in macaque. We fluorescently tagged CBPs in V1 of both species, using antibodies raised against preserved aminoacid sequences. Our data demonstrate important similarities between the expression patterns of interneuron classes in the different layers between rodents and primates. However, in macaque, expression of PV and CB is more abundant, CR expression is lower, and the laminar distribution of interneuron populations is more differentiated. Our results reveal an integrated view of interneuron types that provides a basis for translating results from rodents to primates, and suggest a reconciliation of previous results.

## Introduction

Inhibitory interneurons account for 20 to 30% of cortical neurons (Markram et al. 2004). They play a crucial role in cortical processing (Markram et al. 2004; Isaacson and Scanziani 2011; Fino et al. 2013; Hattori et al. 2017). Interneurons contribute to cortical plasticity (Jones 1993), orientation selectivity (Li et al. 2012), and are also responsible for response normalization (Heeger 1992) and surround suppression (Adesnik et al. 2012).

In macaque, a common scheme to classify interneurons makes use of the calcium-binding proteins (CBPs) parvalbumin (PV), calbindin (CB), and calretinin (CR) (Van Brederode et al. 1990; Condé et al. 1994; DeFelipe 1997; Zaitsev et al. 2005; Disney and Aoki 2008; Kooijmans et al. 2014). In the primary visual cortex, CBP-expressing cells account for approximately 95% of the inhibitory population (DeFelipe et al. 1999; Disney and Aoki 2008). PV is present in chandelier and basket cells, CB in neurogliaform and Martinotti cells, and CR in double bouquet cells. These cell types exhibit specific distributions across the layers (Lund 1987; Lund et al. 1988; Lund and Yoshioka 1991; Lund and Wu 1997) and, accordingly, the expression of CBPs reveals a clear laminar profile (Van Brederode et al. 1990; Disney and Aoki 2008; Kooijmans et al. 2014). Importantly, these specific CBPs are almost exclusively expressed in GABA-ergic cells (Van Brederode et al. 1990; Gonchar et al. 2008; Ma et al. 2013). The same CBPs are also expressed in mouse interneurons (Park et al. 2002; Markram et al. 2004; Burkhalter 2008).

Since CBP-immunoreactive (CBP-IR) morphologies have a high degree of homology across species (DeFelipe 1997; Ascoli et al. 2008; DeFelipe et al. 2013; Hodge et al. 2019), but cortical thickness and complexity differ dramatically (Gilman et al. 2017), a quantitative analysis of CBP-IR populations is essential for comparisons between rodents and primates. A comparative study in the striatum, for example, demonstrated that the distribution of CBP-IR interneurons differs notably between rodents and primates, whereas the expression pattern in the monkey is similar to that in humans (Wu and Parent 2000). A numerical comparison of interneurons classes based on identical protein markers across the cortical layers of mice and monkeys has been lacking.

In the present study, we compared the distributions of inhibitory interneurons in the primary visual cortex between these 2 species, using fluorescent immunohistochemistry for CBPs, and an automated counting technique. As with other architectonic and functional aspects (Laramée and Boire 2014), we observed important similarities between mouse and macaque, and also a few relevant differences. Specifically, for macaque, we found 1) an increase in cells expressing either only PV or only CB, 2) a decreased prevalence of cells expressing only CR, 3) fewer cells coexpressing CBPs, and 4) a more spatially segregated distribution of PV-IR and CB-IR cells across the different sublayers of V1. Furthermore, our results confirm the higher prevalence CBP-IR inhibitory interneurons in primates than in mice (DeFelipe 2011; Džaja et al. 2014). We also compared the size of cell bodies and observed subtle differences between species, including a larger size of PV-IR cell bodies and a smaller size of CB-IR cell bodies in macaque. We expect these findings will be useful for the interpretation of data on mouse interneurons and for the translation of results in mice to nonhuman primates and humans.

## Materials and Methods

### Fixation and Sectioning

All procedures complied with the NIH Guide for Care and Use of Laboratory Animals (National Institutes of Health, Bethesda, Maryland) and were approved by the institutional animal care and use committee of the Royal Netherlands Academy of Arts and Sciences. We used samples from 2 adult male macaque monkey (*Macaca mulatta*) brains, monkeys A and R, and 2 adult male mice (C57BL/6), no 1 and 2. All animals were euthanized and perfused transcardially, first with phosphate-buffered 4% formaldehyde solution, followed by buffered 5% sucrose, both with pH 7.6, at room temperature. After extraction, the brains were placed successively in 12.5% and 25% phosphate-buffered sucrose solutions, at 4 °C, until equilibrium, to prevent subsequent cryo-damage. The macaque brains were then grossly sectioned; from each brain we extracted the entire right occipital lobe posterior to the lunate sulcus, and sagittally split it into 2 equal blocks, to allow for efficient freezing and later cryostat sectioning. All brain samples were then shock-frozen and stored at −80 °C. Several weeks later, we cut the complete macaque occipital samples (2 blocks per specimen) into 20 μm-thick sagittal sections on a sliding freezing microtome. We sectioned the mouse samples coronally (spanning the full antero-posterior extent of V1), guided by documented stereotactic coordinates and anatomical markers (Paxinos and Franklin 2004). We stored the sections for several weeks at −20 °C in 50% glycerol in buffer. We subsequently stained them free-floating.

We performed systematic random sampling of the successive sections by arranging them from medial to lateral (for macaque) and caudal to rostral (for mouse) and dividing them into 4 groups with an equal number of sections. We generated a random number smaller than the group size, and selected the corresponding serial sections from all 4 groups to ensure that every condition covered the same extent of V1.

### Immunohistochemistry

We fluorescently tagged the 3 CBPs (PV, CB, and CR) using antibodies raised against characteristic aminoacid sequences preserved between mouse and macaque. The procedure was performed on 4 sections (selected as explained above), per monkey (A and R), and mouse (1 and 2) with a total of 16 analyzed sections.

**Figure 1:**
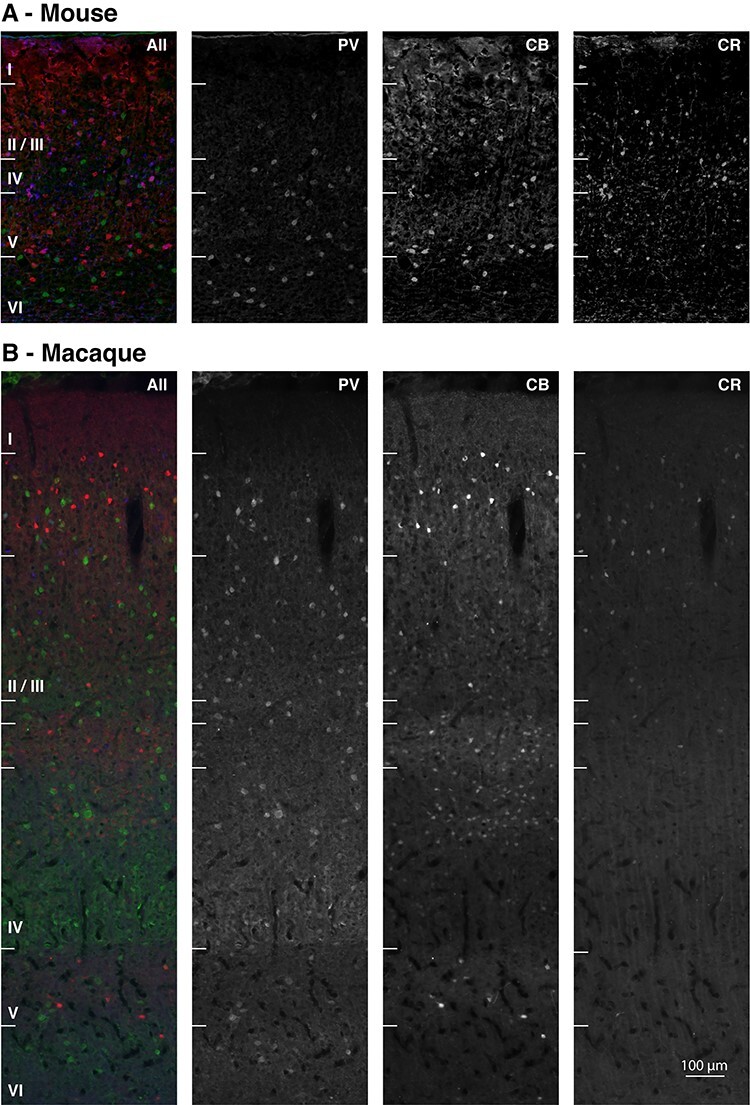
Laminar profiles of parvalbumin (green), calbindin (red), and calretinin (blue) immunoreactivity. The panels represent epifluorescence images from triple-stained 20 μm-thick V1 section of mouse (**A**) and macaque (**B**). The compared layer compartments (I, I/III, IV, V, and VI) are labeled for both species. For macaque, further borders are marked: the transition between layers II and III, as well as the subdivisions of layer IV (IVA, IVB, and IVC). Scale bar is 100 μm.

We removed the sucrose and glycerol protection with 4 rinses in 100 mM phosphate-buffered saline (PBS) at pH 7.6, and blocked unspecific reactivity with donkey serum in PBS (Jackson ImmunoResearch, INC 017-000-121|2:100) with NaN3 (Sigma-Aldrich® 26 628-22-8|1:10000) and Triton™X-100 (Sigma-Aldrich®|1:1000) for 2 h at room temperature. We subsequently incubated the sections overnight with normal donkey serum (5:100), NaN3 and Triton™X-100 and the rabbit primary antibody AB5054 (Merck Millipore: Chemicon| 1:2000) for CR-IR cells. The following day, we removed the primary antibody solution with 2 PBS rinses and incubated with an untagged F (ab^I^)_2_ fragment IgG (H + L) antibody (Jackson ImmunoResearch, INC 111–006-003|1:100) against the shared rabbit host of anti CB and CR antibodies, for 1 h at 36 °C, to differentiate between the targets of the fluorescent secondary antibodies. After another 2 PBS rinses, we incubated the sections overnight, at room temperature, with the remaining primary antibodies, mouse 235 (SWANT® | 1:2000) for PV-IR cells and rabbit CB 38 (SWANT®|1:2000) for CB-IR cells. All primary antibodies used were raised against aminoacid sequences preserved between mouse, macaque, and human (The UniProt Consortium 2010), and have been previously tested for specificity in mouse. These antibodies have been used in previous macaque studies (Morrow et al. 2007; Gonchar et al. 2008; Mascagni et al. 2009; Tricoire et al. 2011; Kooijmans et al. 2014). On the third day, we incubated the sections with their corresponding fluorescent secondary antibodies for 1 h at 36 °C. We used the AlexaFluor® 488 (Molecular Probes® Invitrogen™ A-21202| 1:500) for PV (green channel in Fig. 1), AlexaFluor® 555 (Molecular Probes® Invitrogen™ A-31572| 1:500) secondary antibody for CB (red channel in Fig. 1), and the AlexaFluor® 633 (Molecular Probes® Invitrogen™ A-21082|1:500) secondary antibody for CR stains (blue channel in Fig. 1). We mounted the sections on glass slides, coverslipped them with the Vectashield™ cover medium and preserved them in the dark at 4 °C before imaging.

To control for unspecific staining, we repeated the entire procedure but omitted the primary antibodies and found no fluorescent staining. To test the efficiency of the untagged F (ab^I^)_2_ in separating the signal of the same-host primary antibodies, we carried out the staining procedure including the F (ab^I^)_2_ incubation, and then stained with a fluorescent antibody against the host of the primary antibody. We found no fluorescent signal in this control condition. To validate the specificity of the antibodies, we carried out absorption controls, preincubating the primary antibodies overnight at 4 °C with the corresponding control peptide, as previously documented (Kooijmans et al. 2014), at 10: 1 peptide to antibody molar concentration, before performing the procedure described above, and also observed no fluorescent signal.

### Imaging and Image Processing

**Figure 2:**
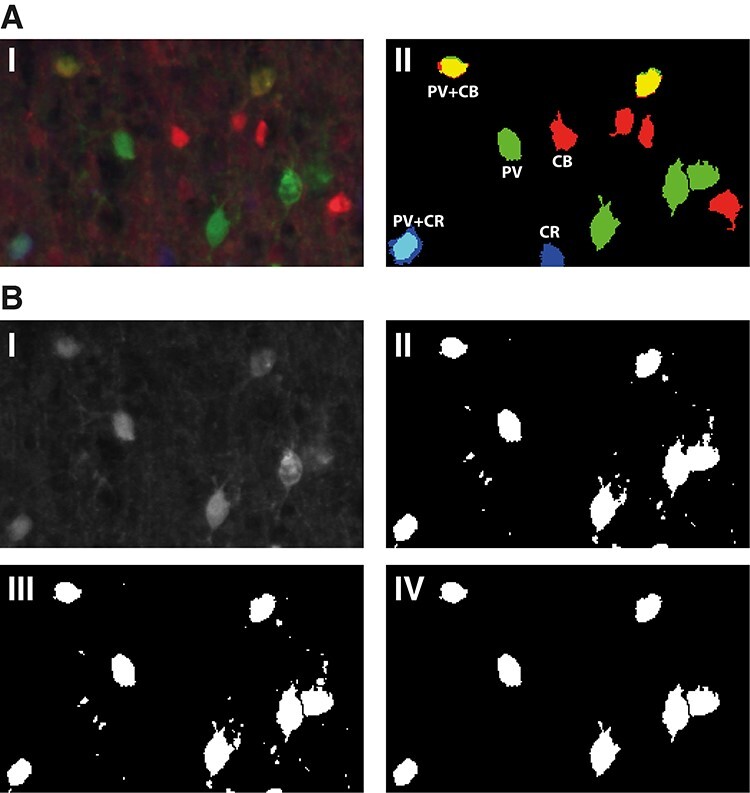
Image processing steps. **A.** I. Detail of layer II/III from a fluorescent 3-channel image of a macaque V1 20μm thick section, stained for PV (green), CB (red), and CR (blue). Note the presence of double-stained cell bodies (cyan:PV + CR, yellow:PV + CB). II. Thresholded 3-channel image with the cell bodies included in the quantification. **B.** Objective thresholding steps for every image channel, illustrated for the green (PV) channel of image A. I. Raw signal; II. Thresholded image with optimal threshold; III. Segmentation of adjoining cell bodies using watershed (see Methods). IV. Cell bodies included in the analysis after thresholding for size (100–500 pixels) and circularity (0.5 to 1).

We acquired 8-bit three-channel RGB images (1392 × 1040pixels) of every section using a Leica DMRD microscope, with a ×10 magnification objective. For the macaque sections, we imaged the center of the outer curvature of the (right) occipital block, in the sagittal direction. For the mouse sections, we imaged the center of the V1 extent in the coronal direction of each section from the (right) occipital lobe. For each section, we sequentially photographed the section using the filter cubes L5 (excitation BP480/40; dichroic 505; emission BP527/30) for AlexaFluor® 488 (PV, green in Figs 1 and 2), Y3ET (excitation BP543/30; dichroic 570; emission BP610/75) for AlexaFluor® 555 (CB, red in Figs 1 and 2) and Y5ET (excitation BP620/60; dichroic 660; emission BP700/75) for AlexaFluor® 633 (CR, blue in Figs 1 and 2). We cropped the acquired images at the pial surface and white matter for each section (for both mouse and macaque, see Fig. 1), with a constant width of 1020 pixels, to facilitate normalization.

For the macaque CB stain, we first excluded the low-intensity cell bodies from further analysis, due to their reported putatively excitatory properties (Van Brederode et al. 1990). Specifically, we subtracted 100 luminance units from the image and thereby completely removed the cell bodies of low intensity. We did not observe a comparable population of weakly stained CB-positive neurons in the rodent, in accordance with previous work (Gonchar and Burkhalter 1997; Park et al. 2002).

We adapted an objective method (Wouterlood et al. 2008; Beliën and Wouterlood 2012; Kooijmans et al. 2014) to count cell bodies (Fig. 2). The method determines a luminance threshold to separate signal pixels from background pixels in each channel of each image. The method sequentially thresholds each image at all possible values (0 to 255), separates aggregated particles using the “Watershed” ImageJ function, and finally applies the “Analyze Particles” function, with a size constraint of 50 to 500 pixels and a 0.5 to 1 circularity constraint and chooses the objective threshold value that yields the highest cell body count. Positive structures that are not cell bodies were automatically discarded using the size and circularity selection criteria (compare panels III and IV in Fig. 2B). We verified that the outcome of this objective and quantitative method were in accordance with our qualitative assessment of colocalization when inspecting the images visually (compare panels I and II in Fig. 2A).

To identify costained cell bodies, we performed a series of basic image calculations. When subtracting binary images, we removed the common signal in the 2 images, while preserving unique signal from the source image, and discarding signals in the subtracted image. We recorded the x/y coordinates of each cell body for every CBP expression profile. We normalized the cortical depth by assigning 0 to the white matter boundary and 1 to the pial surface. For mapping cortical depth, we used 20 bins for macaque, and 10 bins for mouse cortex, so that every bin corresponded to ~ 100 μm, similarly to Van Brederode et al. (1990) and Condé et al. (1994). We also identified layer boundaries, and matched these to the binned data. We counted the total number of cells, the cells in each layer, as well as the cells in each bin. For the binned prevalence data, we normalized the counts to the total number of cells counted in the respective animal. We also recorded the size of each counted cell in pixels. We then derived the cell diameter by approximating a circular shape for CBP-IR interneurons (Van Brederode et al. 1990).

## Results

We compared the expression of CBPs in the different layers of primary visual cortex between mouse and macaque. We observed a pattern of CBP expression that is similar to what was previously described for both mouse (Park et al. 1999, 2002; Gonchar et al. 2008; Xu et al. 2010; Rudy et al. 2011) (Fig. 1A) and macaque (Van Brederode et al. 1990; Disney and Aoki 2008; Ma et al. 2013; Kooijmans et al. 2014) (Fig. 1B). We aimed to go beyond these previous studies, by providing a systematic comparison between the 2 species with a single quantitative and objective method for counting and measuring CBP-IR interneurons continuously, for the entire cortical depth, as well as in the different layers.

### Cell Counts and Densities of Inhibitory Interneurons in Mouse and Macaque

We counted a total of 770 CBP-IR cell bodies in mouse (388 in mouse 1 and 382 in mouse 2) and 3227 in macaque (1526 in Monkey A and 1701 in Monkey R). Even if we take the increased depth of the macaque cortex into account, we observe an increase of 78% in the abundance of inhibitory neurons in the macaque as compared with the mouse. This result is in line with previous reports of higher total neuron density (Herculano-Houzel et al. 2006; Herculano-Houzel et al. 2007; Herculano-Houzel et al. 2015) as well as higher density of inhibitory interneurons (DeFelipe 2011; Džaja et al. 2014) in primates than in rodents. The approximate doubling of the cortical thickness combined with a 78% increase of the interneuron density implies that the number of interneurons below 1 mm^2^ of V1 cortical surface in the monkey is several times larger than the number of interneurons below 1 mm^2^ surface of mouse cortex.

### Relative CBP-IR Cell Frequencies

**Figure 3:**
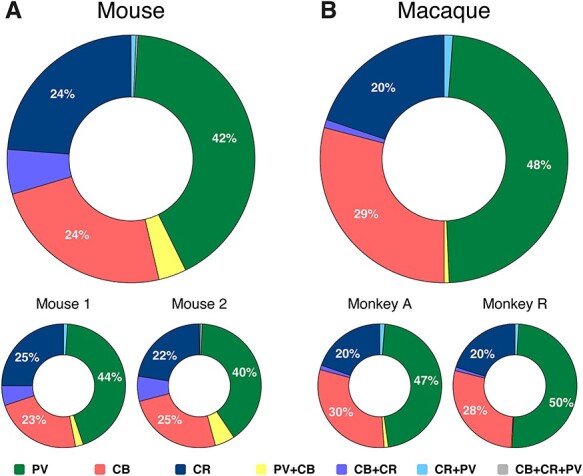
Overall CBP prevalence in mouse (**A**) and monkey (**B**). Percentage of somata in each species (upper panels) and specimen (lower panels). PV:green, CB:red, CR:blue, CB + CR:purple, CB + PV:yellow, CR + PV:cyan, CB + CR + PV:gray.

We first compared the overall distribution of the different interneuron types between species (total cell counts). We found that the expression of CBPs in inhibitory interneurons was similar in mouse and monkey (Fig. 3A, B). In mouse, 45% of the counted interneurons were positive for PV, 33% for CB, and 30% for CR (these numbers add up to more than 100% because some cells were doubly labeled). In monkey, 49% of the cells were positive for PV, 31% for CB, and 22% for CR; a higher count for PV complemented by a lower CR count in monkey, as compared with mouse. The results were similar for individual specimens from the same species (Fig. 3).

The majority of segmented neurons (89.7% in mouse and 97.1% in macaque) expressed a single CBP, with 42% PV-only, 24% CB-only, and 24% CR-only interneurons in the mouse, and 48% PV-only, 29% CB-only, and 20% CR-only cells in the macaque (see also van Brederode et al. 1990). We observed a small population (10.3% in mouse and 2.9% in macaque) coexpressing multiple CBPs (Fig. 3A, B), as previously documented in mouse (Park et al. 2002; Gonchar et al. 2008) and macaque (Van Brederode et al. 1990; Härtig et al. 1996; Sherwood et al. 2007). In mouse, the largest coexpressing fractions were CB + CR with 5.8% and CB + PV with 3.7% of cells. The fractions of neurons that coexpressed CR + PV (0.63%) or CB + CR + PV (0.2%) were much lower. In macaque, the fraction of cells positive for CB + CR and CB + PV were 1.1% and 0.7%, respectively, 5 times less than in mouse (Fig. 3). The fraction of neurons positive for CR + PV was 1.2%, which is twice as large as in mouse. We found only a single triple stained neuron (CB + CR + PV) in the 2 macaques (0.03%).

### Distribution of Interneuron Classes across the Cortical Layers

**Figure 4:**
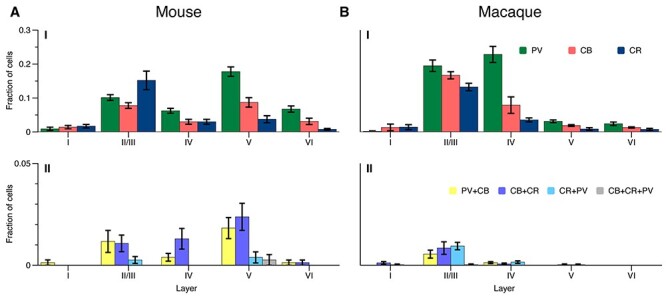
Average CBP-IR cell counts per layer in mouse (**A**) and macaque (**B**), normalized to the overall cell count. Bars represent standard error of the mean across sections. PV:green, CB:red, CR:blue, CB + CR:purple, CB + PV:yellow, CR + PV:cyan, CB + CR + PV:gray.

We next analyzed the number of interneurons of the various classes in layers I, II/III, IV, V, and VI, normalizing the cell counts to the total number of cells per animal (Fig. 4: values represent the average fraction of cells). We selected this layer partition, standard for mouse, as a common denominator between the 2 species’ cortical complexity. Macaque cortex presents more complex, previously documented sublamination (Van Brederode et al. 1990; DeFelipe et al. 1999; Disney and Aoki 2008; Kooijmans et al. 2014), as is also indicated in Figures 1 and 6, and covered in the continuous mapping and discussion. This complexity however does not have a comparable equivalent in mouse, and was therefore incorporated into the overarching corresponding compartments, to allow for direct statistical analysis.

Both species had a similar CR-IR expression profile across the layers, with the highest CR-IR proportion layer II/III (Fig. 4), as previously observed in mouse (Park et al. 2002) and macaque V1 (Disney and Aoki 2008; Ma et al. 2013). In mouse layer II/III CR-IR neurons outnumbered CB-IR cells, but in layer V there were more CB-IR than CR-IR cells (Fig. 4A), as reported by Park et al. (2002). The CB-IR and PV-IR cell body distributions had a more complex pattern across the layers (Fig. 4A, B). In mouse, PV-IR cell counts were highest in layers II/III and V. In macaque, the highest PV cell counts were in layers II/III and IV, similar to the results of van Brederode et al. (1990), and PV expression was much lower in layer V.

We calculated a 3-way analysis of variance (ANOVA) (species x layer x CBP) to assess the existence of a systematic species-driven effect in the data, and found no main effect of species, but a significant main effect of layer (F (4, 210) = 90, *P* < 0.001) and of CBP (F (2, 210 = 36, *P* < 0.001). We also found a significant interaction effect between layer and species (F (4, 210) = 36, *P* < 0.001), a significant interaction effect between layer and CBP (F (8, 210) = 9.2, *P* < 0.001), as well as a significant 3-way (species x layer x CBP) interaction (F (8, 210) = 10.7, *P* < 0.001). These results suggest that the interspecies effect is driven by the arrangement of CBPs across different layers, as will be described in the rest of our analysis. We then calculated a 2-way (layer x CBP) ANOVA, per species, to test the statistical significance of the observed differences in cell prevalence. For mouse, both main effects of layer (F (4,105) = 28), and CBP (F (2,105) = 10.8) as well as the interaction effect (F (8,105) = 7.2) were significant, with all *P*s < 0.001. The same was true for macaque with F (4,105) = 117 for layer, F (2,105) = 32.1 for CBP, and F (8,105) = 14.2 for the interaction effect, and all *P*s < 0.001.

We did not perform a cross-layer statistical analysis for the cells expressing multiple CBPs due to the large number of zero values in our samples 320 samples (2 species × 2 specimens × 4 sections × 5 layer-locations × 4 coexpressions; Fig. 4). In mouse, coexpressing cells (10.3% of all counted cells) were mostly located in layers II/III to V (Fig. 4A), as previously reported (Park et al. 2002). In macaque, the small number of coexpressing cells (2.9% of all counted cells) were mostly located in layer II/III (Fig. 4B) (Van Brederode et al. 1990; Leuba et al. 1998).

### Prevalence of CBP-IR Neurons within the Cortical Layers

**Figure 5:**
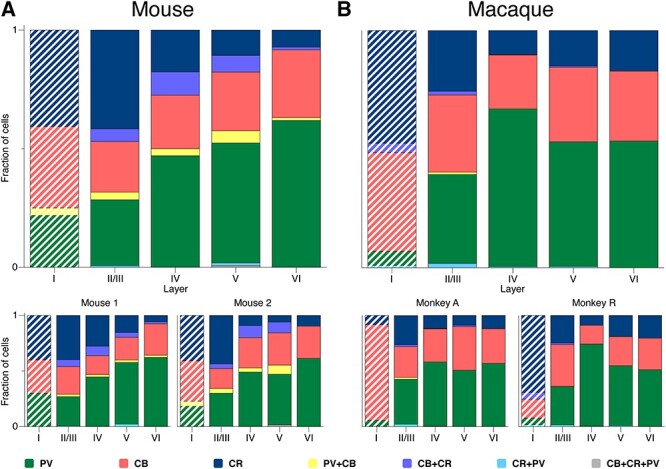
CBP-IR fraction within the cortical layers of mouse (**A**) and macaque (**B**) V1. PV:green, CB:red, CR:blue, CB + CR:purple, CB + PV:yellow, CR + PV:cyan, CB + CR + PV:gray. Hatched bars: data for layer 1 was variable across sections.

We next determined the fraction of interneurons labeled for single and multiple CBPs within each layer (total cell bodies counted; Fig. 5A, B). Neuronal cell-bodies in layer I were few (<3%) and variable in CBP expression in our sample (data of individual monkeys are shown in Fig. 5B), so we do not draw proportional conclusions from the data in layer I.

We found that the percentage of PV-IR cells increased with laminar depth in mouse (Fig. 5A). The laminar pattern in macaque differed, with the highest percentage of PV-cells in layer IV (Fig. 5B). The opposite held for CR-IR cells. Their proportion decreases with laminar depth in both mouse and macaque. The fraction of CB-IR cells was relatively stable across the layers.

### Continuous Mapping of CBP-IR Neurons along the Cortical Depth

**Figure 6:**
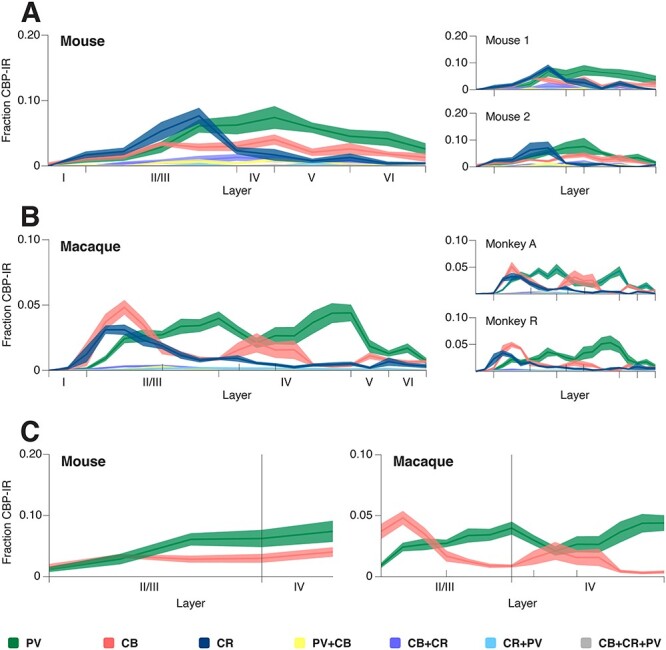
CBP laminar profile in mouse (**A**) and macaque (**B**) as function of cortical depth. **C**. Detail of CB-IR and PV-IR sublaminar profiles for layers II/III and IV in mouse and macaque. Lines represent averages, shaded areas—standard error of the mean. The compared layer compartments (I, I/III, IV, V, and VI) are labeled for both species. For macaque, further borders are marked: the transition between layers II and III, as well as the subdivisions of layer IV (IVA, IVB, and IVC).

For our next analysis, we omitted cell body preassignment to specific cortical layers. Instead, we determined the relative location of every interneuron on a continuous scale between the white matter boundary and the pial surface (see Methods). We then defined bins of approximately 100 μm, with 20 bins for the macaque cortex and 10 bins for the mouse (Fig. 6). In each bin, we normalized the PV-IR, CB-IR, and CR-IR cell counts to the total number of CBP-IR cells per animal. The total number of cells expressing the labeled CBPs was similarly distributed across the cortical depth in both mouse and macaque, with a largely unimodal distribution peaking in layer II/III. The expression of CR was similar between the species, with a prominent peak in layer II/III (Fig. 6) (see also Park et al. 2002; Disney and Aoki 2008; Ma et al. 2013; Kooijmans et al. 2014), reflecting the abundance of CR-positive bipolar cells in this layer (Burkhalter 2008; Disney and Aoki 2008; Kooijmans et al. 2014).

The expression profiles of PV and CB differed between mouse and macaque. The mouse distributions for both CBPs were largely unimodal and peaked in layer V, close to the boundary with layer IV (Fig. 6A). The macaque distributions had multiple peaks (Van Brederode et al. 1990), with an anticorrelation between the density of PV-IR and CB-IR populations across the layers (Fig. 6B, C). This arrangement can also be seen in Figure 1B as alternating bands of PV-IR and CB-IR neurons in the different layers. In macaque, the density of PV cells peaked at the boundary between layer II/III and layer IV (IVa), in lower layer IV (layer IVc), and in layer VI (Fig. 6B) (Van Brederode et al. 1990). The CB-IR population peaked in upper layer II/III, had a second, shallower peak in layer IV (a/b), and peaked again in layer V (Fig. 6B). The 2 specimens of each species showed similar expression patterns across the layers (Fig. 6A, B). In order to statistically test the presence of sublayer patterns in layers II/III and IV of the PV-IR/CB-IR populations, we calculated the correlation coefficient between the CB-IR and PV-IR cell counts for each section (8 per species), for the depth corresponding to these layers in both mouse (5 bins) and macaque (14 bins). The correlation coefficients did not differ significantly from 0 in the mouse (one-sample *t*-test, t (7) = 1.1 *P* > 0.3, 95% CI), but were significantly negative in the monkey with an average of −0.34 (95% CI = [−0.46,−0.2], one-sample *t*-test, t (7) = −6.1 *P* < 0.0001). The correlation coefficient was also significantly lower in monkey than in mouse (0.2 ± 0.53, independent sample *t*-test, t (14) = −2.78, *P* < 0.05), supporting the observation that CB-IR and PV-IR cell bodies are in similar layers in the mouse but in alternating bands in the macaque (Figs 1 and 6C).

### CBP-IR Cell (Body) Size in Mouse and Monkey

**Figure 7:**
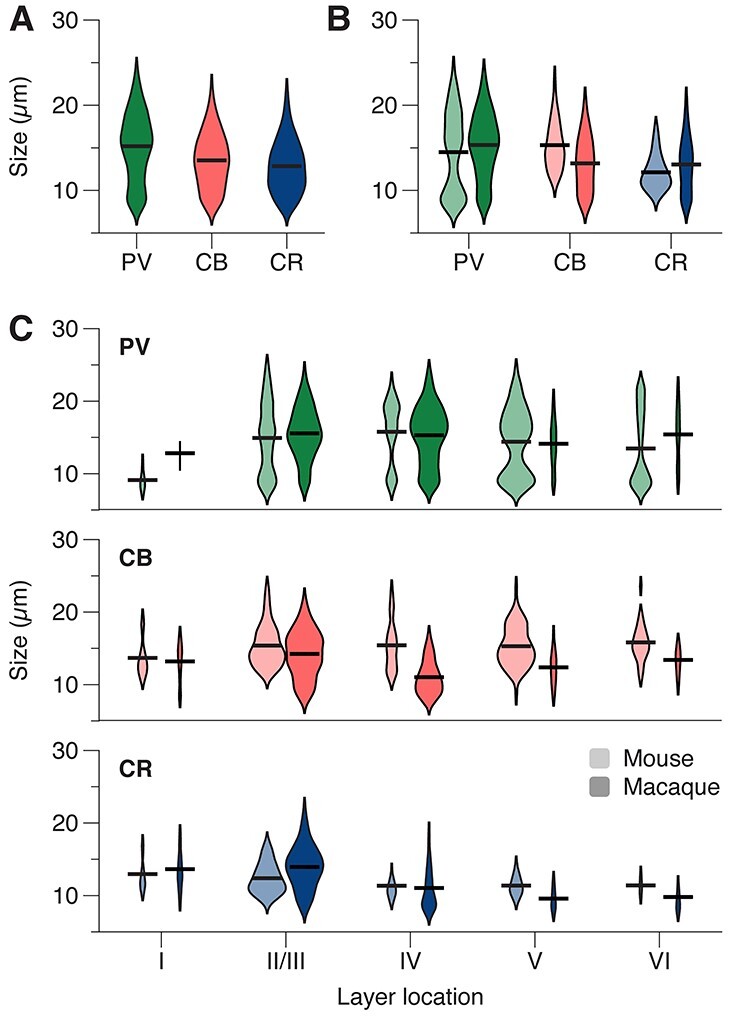
Cell size distribution for CBP-IR populations. **A.** Violin plots of size distributions for cells expressing PV:green, CB:red, CR:blue. **B.** Violin plots of PV-IR, CB-IR and CR-IR cell size distributions for mouse (unsaturated colors) and macaque (saturated colors). **C.** Violin plots of size distributions of PV, CB, and CR-IR cells, per layer. Violin surfaces scale with population fraction corresponding to layer, and are normalized per species and CBP: violin surfaces per CBP add to the same area in each species. The horizontal lines indicate averages.

We considered the possibility that differences in the size of cell bodies might have influenced the results, because larger cell bodies have a higher probability to be detected. We therefore performed a 3-way ANOVA with the factors: species, layer, and CBP (3824 cell bodies; 690 in mouse and 3134 in macaque; Fig. 7A, B). We found no main effect of species (F (1, 3794) = 3.9, *P* = 0.05), indicating no overall size differences between mouse and macaque.

We did observe a significant main effect of CBP (F (2, 3794 = 35, *P* < 0.001; Fig. 7A). PV-IR cells had the largest average size (15.2 μm), followed by CB-IR cells (13.5 μm) and CR-IR cells (12.8 μm) (Fig. 7A; Bonferroni-corrected pairwise comparisons, all *P*s < 0.001). Furthermore, there was a significant difference in the size of cell bodies between layers (main effect, F (4, 3794) = 16, *P* < 0.001).

There were also 2-way interactions between CBP and species (F (2, 3794) = 17.8, *P* < 0.001; Figure 7B), CBP and layer (F (8, 3794) = 6.9, *P* < 0.001), and between layer and species (F (4, 3794) = 8.4, *P* < 0.001), but no significant 3-way interaction (F (8, 3794) = 1.9, *P* = 0.051). To assess how these interactions influenced our findings, we determined cell-body size per species and layer, separately for each CBP. PV-IR cell bodies were slightly larger in the monkey (average diameter of 15.3 μm) than in the mouse (14.5 μm) (F (1, 1866) = 4.6, *P* = 0.03) and this size difference may have provided a small contribution to their slightly higher prevalence in the monkey (Fig. 3). In contrast, CB-IR cell bodies were larger in mice than monkeys (15.3 μm in mice vs. 13.1 μm in monkeys, F (1, 1114) = 54.6, *P* < 0.001) but their prevalence was lower in mice, which can therefore not be explained by a difference in the size of cell bodies. The size of CR-IR cell bodies was similar between species (F (1, 814) = 0.6, *P* = 0.4). In the monkey, CB-IR cell bodies had a particularly small size in layer IV (interaction, F (4, 1114) = 5.996, *P* < 0.001), an effect that is presumably driven by the neurogliaform cell population (Jones 1984; Nieuwenhuys et al. 2007). These results, taken together, indicate that the differences in the prevalence of the different interneuron classes between species were not caused by differences in cell body sizes.

## Discussion

We determined the laminar distribution of CBP-IR interneurons in the primary visual cortex of mouse and macaque. This is the first study to systematically compare these distributions. Our findings mostly agree with previous descriptions of CBP-IR cell distributions (Van Brederode et al. 1990; Park et al. 1999, 2002) but go beyond by providing a systematic between-species comparison, based on an objective and automatic quantification method.

The density of inhibitory interneurons was higher in monkeys than in mice, in accordance with previous work (Lin et al. 1986; Fitzpatrick et al. 1987; Hendry et al. 1987; Meinecke and Peters 1987; Beaulieu et al. 1992; Beaulieu et al. 1994; Hornung and De Tribolet 1994; Jones et al. 1994; Micheva and Beaulieu 1995; Del Río and DeFelipe 1996; Gabbott and Bacon 1996a, 1996b; Gabbott et al. 1997; Tamamaki et al. 2003; Džaja et al. 2014).

We found that the number of cells expressing only CR was lower in the macaque than in the mouse, but that their location was similar. Accordingly, the prevalence of cells expressing only PV or CB was higher in the macaque. Furthermore, the distribution of interneurons across the layers differed with highest counts in layers II/III and V in the mouse and in layers II/III and IV in the macaque (Fig. 4). The shift of highest PV expression in layer V in mouse to layer IV in macaque may be related to the increase in size and complexity of layer IV in the macaque as compared with the mouse (Gilman et al. 2017). Additionally, there was a more complex sublayer pattern in macaque. These data illustrate that there are important similarities, but also some interesting differences between these species, that are most likely caused by the increase in functional complexity in the macaque.

### Methodological Concerns

We conducted a number of checks to ensure the quality of our data. We chose primary antibodies based on previous published data (Methods), with documented specificity, which resulted in an excellent match with previous descriptions of the laminar profile and morphology of the 3 inhibitory cell classes (Van Brederode et al. 1990; Condé et al. 1994; Disney and Aoki 2008; Ma et al. 2013; Kooijmans et al. 2014). We also ruled out cross-reactivity between primary and secondary antibodies (Kooijmans et al. 2014). Furthermore, we used an objective quantitative method to count cell bodies, which allowed for the precise mapping of cell bodies with different CBP expression, with the possibility to normalize and compare across varying sample cortical depth and species. The results of this analysis were in accordance with visual inspection of the data (Fig. 1) and previous partial qualitative and quantitative reports.

We also considered the possibility that the relatively subtle size differences of the cell-bodies might have influenced the cell density estimates, because larger cell-bodies might increase the probability that cells are counted. However, our results are not compatible with this explanation. Firstly, cell-bodies of PV-IR neurons in the monkey were approximately 5% larger than those in the mouse, but the prevalence of PV-IR neurons was 14% higher in the monkey. Secondly, cell-bodies of monkey CB-IR neurons were 17% smaller than those in the mouse but had a 21% higher prevalence. Thirdly, there were no interspecies differences in average cell size of the CR-IR neurons but the proportion of CR-IR neurons was smaller in the macaque. It is therefore unlikely that variations in cell-bodies sizes account for the differences in the prevalence of the interneuron classes between species.

### Comparison with Previous Studies

The expression of PV across the layers is reminiscent of the results of Gonchar et al. (2008) who also identified 2 peaks in PV expression, in layers II/III and V (Fig. 4A, Gonchar et al. 2008—their figure 2). The CR and CB layer counts were in agreement with the data of Park et al. (2002), with 1 peak for CR in layer II/III, and 2 for CB, in layers II/III and V, respectively. Considering the large overlap of CB and SST (Gonchar and Burkhalter 1997; Hladnik et al. 2014), our data are also in general agreement with those of Gonchar et al. (2008) and Rudy et al. (2011).

We observed a prevalence of CR-IR cells in macaque striate cortex similar to that reported by Yan et al. (1995) and Ma et al. (2013), but lower than estimated by Hladnik et al. (2014) for the frontal cortex. A large CR-IR population is present in macaque prefrontal cortex (Condé et al. 1994), whereas V1 has a larger fraction of PV-IR interneurons (Van Brederode et al. 1990; Ma et al. 2013), characterizing primary sensory cortices with an elaborate layer IV (Gilman et al. 2017). The laminar location of CR-IR cell bodies is in agreement with the 1 described by Meskenaite (1997).

The laminar distribution of PV and CB positive interneurons in the monkey is in general agreement with the results by Van Brederode et al. (1990), although their analysis grouped the lower part of layer III with layer IVA. The continuous PV distribution we find is also in line with to the 1 reported by Van Brederode et al. (1990), as well as Kelly et al. (2019). We however report an additional CB-IR peak in layer IVB which is visible in their data, but not significant. The existence of this CB-IR band in layer IVB was reported by subsequent studies (Pinheiro Botelho et al. 2006; Disney and Aoki 2008; Ma et al. 2013; Kooijmans et al. 2014), and presumably corresponds to the layer IV CB-IR neurogliaform cell population, characteristic of primate visual and somatosensory cortices (Jones 1984; Kisvárday et al. Kisvarday et al. 1986, Kisvárday et al. 1990; Nieuwenhuys et al. 2007).

### CBPs Versus Alternative Labeling Schemes

Our main aim was to compare the distribution of CBPs between mouse and macaque. We took advantage of PV, CB, and CR as protein markers whose expression is preserved across multiple species (DeFelipe 1997; Ascoli et al. 2008; DeFelipe et al. 2013; Hodge et al. 2019). There are numerous other markers, associated with specific interneuron populations in mice, which are however not consistently expressed in primates (Hendry et al. 1984; Campbell et al. 1987; Jakab and Goldman-Rakic 2000). PV, CB, and CR are members of the “EF-hand” Ca^2+^-binding protein family, present in numerous cells types (Lewit-Bentley a, Réty S. 2000). CPBs are essential in maintaining Ca^2+^ homeostasis and regulate presynaptic and postsynaptic Ca^2+^ signaling (Schwaller et al. 2002). CB and CR have more EF-hands than PV and are faster Ca^2+^ buffers (Chard et al. 1993; D’Orlando et al. 2002; Schwaller et al. 2002; Grabarek 2006; Barinka and Druga 2010). Hence, a classification scheme based on CBPs could also be relevant from a physiological point of view, because it may relate to the firing pattern of neurons (Zaitsev et al. 2005).

In primates, CBP labeling is standard because of exhaustive (Van Brederode et al. 1990; Disney and Aoki 2008) and largely separate labeling of the interneurons, although small populations that coexpress multiple CBPs exist (Van Brederode et al. 1990; Leuba et al. 1998). In mouse, however, there are several alternative labeling-schemes (Gonchar et al. 2008; Xu et al. 2010; Rudy et al. 2011). A prevalent scheme for labeling in mouse divides inhibitory interneurons into 3 populations: immunoreactive for PV, somatostatin (SST), and the serotonin receptor 5HT3aR (Lee et al. 2010; Rudy et al. 2011). There are a number of additional markers, including as NPY, NOS, ChAT that identify very specific but smaller populations in mouse (Markram et al. 2004; Gonchar et al. 2008). PV is a preserved marker in both labeling schemes and it reliably labels fast-spiking inhibitory neurons. In contrast, SST and 5HT3aR label only few cell-bodies in the macaque (Hendry et al. 1984; Campbell et al. 1987; Jakab and Goldman-Rakic 2000), and are therefore not useful cross-species markers. New methods, which define cell types at a transcriptomic level, offer new insights into inhibitory cell type diversity and ontology (Lim et al. 2018; Favuzzi et al. 2019; Hodge et al. 2019). CBP expression gives complementary insights by facilitating the direct comparison of interneuron class distributions that correlate with morphology.

### Complementary PV and CB Distribution and Function

The results of the analysis based on the relative distances between a cell body and the pial and white matter were largely compatible with the results when first assigning them to the different layers. There was 1 exception, however. When we continuously mapped cell bodies along the cortical depth, we encountered a complementary, band-like arrangement, with peaks in PV density aligning with troughs in CB density, and vice versa. ln layer II/III, the PV-IR population is spatially distinct from the CB-IR population (Van Brederode et al. 1990), with a CB-IR peak in upper layer II, followed by a PV-IR peak in lower layer III, which extends into upper layer IV (layer IVA; Fig. 6B) (Van Brederode et al. 1990). This is followed by a CB-IR peak in intermediate layer IV (layer IVB) (Pinheiro Botelho et al. 2006; Nieuwenhuys et al. 2007), and finally a PV-IR peak in lower layer IV (layer IVC) (Van Brederode et al. 1990; Disney and Aoki 2008; Kooijmans et al. 2014). This PV/CB alternating peak pattern reflects the intricate sublayer complexity in the macaque (Fitzpatrick et al. 1985; Lund 1987; Lund and Yoshioka 1991), which is absent in the mouse. Additional to their anticorrelated arrangement, PV-IR and CB-IR neurons (CB-IR overlap with SST-IR cells; Gonchar and Burkhalter 1997; Hladnik et al. 2014) are complementary in their function/connectivity (Adesnik et al. 2012), firing pattern (Zaitsev et al. 2005), and AMPA receptor expression (Kooijmans et al. 2014).

### Lateral Variation

There are likely interspecies differences in the distribution of interneurons in the tangential cortical direction. For example, cytochrome oxidase blobs in layers II/III of *Cebus apella* monkeys are enriched in PV relative to the interblob regions (Pinheiro Botelho et al. 2006) but cytochrome oxidase does not appear to delineate comparable organization of layer 2/3 in mouse V1 (Laramée and Boire 2014). However, PV enrichment has been reported to be associated with muscarinic acetyl-choline receptor patches in layer I in mouse (Ji et al. 2015; D’Souza et al. 2019). We however focused on the distribution of interneurons along the cortical depth, and sampled blind to the aforementioned modularity. The cross layer/depth-profile variability appears to override horizontal variability for PV-IR cells (previously investigated), as it renders highly significant results in our analysis. This also holds true for each of the other CBPs, as well as their comparative profiles. Future studies will therefore be needed to characterize possible differences in the tangential interneuron distribution between these species.

## Summary

Our results show comparable overall expression patterns and sizes of CBP-IR cell bodies in the primary visual cortex of mice and monkeys. We also report a number of notable differences between species. We found 1) complementary PV-IR and CB-IR sublayer expression patterns in the macaque, which were not seen in the mouse and 2) a shift of PV-IR interneurons from mouse layer V toward macaque layer IV. We expect that the present and future comparisons of the anatomy and function of the cerebral cortex between mice and primates will facilitate the translation of the rapidly growing insights into the function of mouse cortex to primates and, ultimately, to humans.

## Notes

We thank Kor Brandsma, Corbert G. van Eden, Jan Klooster, and Chris Pool for biotechnical assistance. *Conflict of Interest*: None declared.

## Funding

The work was supported by an NWO Brain and Cognition program grant (433-09-208), the European Union Seventh Framework Program (ERC Grant Agreement 339490 “Corticalgorithms”), as well as H2020 grant agreements 720270 and 785907 “Human Brain Project SGA1 and SGA2”, and the Friends Foundation of the Netherlands Institute for Neuroscience awarded to P.R.R.
